# Plasma Myokine Concentrations After Acute Exercise in Non-obese and Obese Sedentary Women

**DOI:** 10.3389/fphys.2020.00018

**Published:** 2020-02-18

**Authors:** Léa Garneau, Stephanie A. Parsons, Steven R. Smith, Erin E. Mulvihill, Lauren M. Sparks, Céline Aguer

**Affiliations:** ^1^Institut du Savoir Montfort, Ottawa, ON, Canada; ^2^Department of Biochemistry, Microbiology and Immunology, Faculty of Medicine, University of Ottawa, Ottawa, ON, Canada; ^3^Translational Research Institute for Metabolism and Diabetes, AdventHealth Orlando, Orlando, FL, United States; ^4^Energy Substrate Laboratory, University of Ottawa Heart Institute, Ottawa, ON, Canada; ^5^School of Human Kinetics, Faculty of Health Sciences, University of Ottawa, Ottawa, ON, Canada

**Keywords:** myokines, exercise, interleukins, FGF21, secreted protein acidic rich in cysteine, training, obesity, skeletal muscle

## Abstract

Exercise and physical activity levels influence myokine release from skeletal muscle and contribute to circulating concentrations. Indeed, many myokines, including interleukin (IL)-6, IL-15, secreted protein acidic rich in cysteine (SPARC), and fibroblast growth factor (FGF) 21 are higher in the circulation after an exercise bout. Since these peptides modulate muscle metabolism and can also be targeted toward other tissues to induce adaptations to energy demand, they are of great interest regarding metabolic diseases. Therefore, we set out to compare, in six women with obesity (BMI ≥30 kg/m^2^) and five healthy women (BMI 22–29.9 kg/m^2^), the effect of an acute bout of moderate-intensity, continuous cycling exercise (60 min, 60% VO_2_peak) on the release of myokines (IL-6, IL-8, IL-10, IL-13, IL-15, SPARC, and FGF21) in plasma for a 24-h time course. We found that plasma IL-8 and SPARC levels were reduced in the group of women with obesity, whereas plasma IL-13 concentrations were elevated in comparison to non-obese women both before and after the exercise bout. We also found that plasma FGF21 concentration during the 24 h following the bout of exercise was regulated differently in the non-obese in comparison to obese women. Plasma concentrations of FGF21, IL-6, IL-8, IL-15, and IL-18 were regulated by acute exercise. Our results confirm the results of others concerning exercise regulation of circulating myokines while providing insight into the time course of myokine release in circulation after an acute exercise bout and the differences in circulating myokines after exercise in women with or without obesity.

## Introduction

Since the beginning of the 2000s, skeletal muscle has gained notoriety as an important source of secreted factors such as peptides, RNAs, metabolites, and extracellular vesicles that can contain any of these molecules. These factors are significant regulators of whole-body energy metabolism. The peptides secreted by the skeletal muscle are called myokines, as many of these peptides are also cytokines [e.g., the interleukins (IL)] (Pedersen et al., 2003). Myokines have been studied not only for their effect on skeletal muscle metabolism but also as endocrine effectors of energy metabolism adaptions (Nielsen and Pedersen, 2007; Garneau and Aguer, 2019).

During exercise and/or muscle contraction, the levels of some myokines in the muscle are greatly increased, and circulating levels of these peptides are also modulated by exercise. For example, after an acute bout of moderate-intensity cycling on a stationary bicycle (70% VO_2_max), IL-6 and fibroblast growth factor (FGF) 21 expression was increased in skeletal muscle, while serum levels of IL-6, IL-15, and FGF21 were significantly increased (Sabaratnam et al., 2018). The effect of exercise on the secretion of myokines from skeletal muscle locally cannot necessarily be translated to the release of these peptides in the circulation, as reviewed in Garneau and Aguer (2019). Indeed, some myokines are believed to act directly on skeletal muscle to improve its energy metabolism during contraction. For example, IL-6 increases muscle fatty acid oxidation and glucose uptake in L6 myotubes and human primary muscle cells from *rectus abdominis* muscle (Al-Khalili et al., 2006; Carey et al., 2006), while IL-13 treatment directly stimulates glucose uptake and oxidation in human primary myotubes (Jiang et al., 2013). On the contrary, the mechanism of action of myokines can also require their release in the circulation to be delivered to their target tissue(s). Examples of such mechanisms include the stimulation of fatty acid oxidation and glucose uptake into white adipose tissue by IL-6 during exercise recovery (Knudsen et al., 2017) and the positive effect of IL-13 on both rat and human pancreatic β-cell survival (Rutti et al., 2016). This underscores the different mechanisms of action of myokines depending on their release locally in skeletal muscle [muscle protein and messenger RNA (mRNA)] and in the circulation (plasma or serum protein). The metabolic functions of myokines draw a link between exercise adaptations and amelioration of muscle and whole-body metabolisms, which is of interest in the context of obesity, as it is an important risk factor for the development of type 2 diabetes (T2D) (Muoio and Newgard, 2008).

In patients affected with certain chronic non-communicable diseases (e.g., sarcopenia, arthritis, obesity, and T2D), circulating myokine levels are altered. These diseases are generally associated with systemic, low-grade inflammation (Gregor and Hotamisligil, 2011). For example, IL-6 and FGF21 were found to be elevated in patients with obesity and correlated with whole-body adiposity (Vozarova et al., 2001; Zhang et al., 2008); circulating IL-8 and secreted protein acidic rich in cysteine (SPARC) correlated positively with BMI (Kim et al., 2006; Wu et al., 2011); while plasma IL-15 not only correlated negatively with trunk fat mass and body-fat percentage (Nielsen et al., 2008) but have also been shown to be elevated in the setting of obesity (Perez-Lopez et al., 2018). It is well-established that acute and chronic exercise can modulate circulating myokine levels. However, it is currently unclear if the presence of obesity (BMI >30 kg/m^2^) can impact the myokine response in sedentary subjects. In this regard, our study design allows us to observe the potential effects of obesity on the variations in circulating myokines following an acute bout of exercise.

Here, we evaluate the dynamic and time-dependent changes in plasma myokine concentrations [pre-, immediately after an acute bout of moderate-intensity cycling exercise (0 h), 1, 2, 3, 4, 12, and 24 h postexercise] in sedentary women with and without obesity.

## Materials and Methods

### Participants and Study Design

A total of 11 women, 18–40 years of age, were included in this study; six women were classified as obese (BMI >30 kg/m^2^), and five were classified as non-obese (BMI = 22–29.9 kg/m^2^). Other inclusion and exclusion criteria were as follows: weight stable (<3 kg variations in the last 8 weeks); not involved in any exercise program; willing to stop caffeine and alcohol consumption 48 h before blood draw; no history of T2D; glycated hemoglobin (HbA1c) ≤ 6.5% (measured in the obese group only); favorable anatomy for continuous venous blood sampling; no presence of clinically significant abnormalities on ECG; no significant renal, cardiac, liver, lung, or neurological disease (controlled hypertension acceptable); no use of drugs known to affect metabolism or body weight (e.g., orlistat, sibutramine, ephedrine, phenylpropanolamine, corticosterone); current treatment with blood thinners or antiplatelet medications that cannot be stopped safely; no new onset (<3 months) of oral contraceptives or hormone replacement therapy; not current smokers (>3 months); not currently pregnant or having nursed a child within the last 12 months; no gait problems; no increased liver function tests (aspartate aminotransferase/alanine aminotransferase/gamma-glutamyl transferase or alkaline phosphatase >2.5 times the upper limit of normal); no in-body metal objects that would interfere with body composition measurements; no New York Heart Association any class heart failure; no history of deep vein thrombosis or pulmonary embolism; no significant varicose veins; no abnormal blood count or blood donations in the last 2 months; no major surgery of the abdomen, pelvis, or lower extremities in the last months; no bariatric surgery or liposuction within the previous 3 years; no cancer; no rheumatoid disease; no bypass graft in limb; no known genetic factor (e.g., Factor V Leiden) or hypercoagulable state; no diagnosed peripheral arterial or vascular disease or intermittent claudication; no peripheral neuropathy; no claustrophobia; no major depression; no presence of an eating disorder or eating attitudes/behaviors that could interfere with the study; and no nocturnal urination and/or sleep apnea.

Two days before performing the exercise bout, participants underwent a diet stabilization period and were given standard diet composition meals (35% fat, 15% protein, and 50% carbohydrates; prepared and prepacked at the exercise facility). The day before the exercise test, participants came fasted at the facility, underwent all baseline measures, then ate the standardized diet for breakfast and lunch. At night, they consumed a high-carbohydrate dinner and snack (70–75% carbohydrate, 12–15% fat, 10% protein), then performed the cycling exercise bout at around 7 AM the following morning. Plasma samples from these participants were obtained immediately before (pre-), immediately after (0 h), and 1, 2, 3, 4, 12, and 24 h into the recovery period after a fasted acute bout of moderate-intensity continuous exercise. The exercise bout consisted of 60 min of cycling on an upright stationary bicycle (Vision Fitness U40, Wisconsin, United States) at 60% VO_2_peak. Participants remained fasted for the first 4 h of recovery with limited liquid for consumption (600 ml) and were fed meals of standard diet composition (35% fat, 15% protein, and 50% carbohydrates) after the “4 h” blood draw, with limited total liquid consumption of 1,000 ml. Blood samples were collected with a venous catheter in standard anticoagulant (ethylenediaminetetraacetic acid) tubes, centrifuged at 2,000 × *g* for 10 min, aliquoted, and stored at −80°C until analyses.

### Body Composition, Blood Analyses, and VO_2_max Determination

Whole-body fat and lean mass were assessed through dual energy X-ray absorptiometry scans (GE Lunar iDXA whole-body scanner, GE Healthcare, United States). Glucose, insulin, and HbA1c were measured in fasted blood samples in the clinical chemistry laboratory at either Florida Hospital or onsite at the Translational Research Institute for Metabolism and Diabetes, as previously described (Stephens et al., 2018) to assess the eligibility of the participants. An HbA1c ≥ 6.5% would have resulted in the exclusion of the participant from the study. Glucose and insulin levels were used to determine homeostatic model assessment of insulin resistance (HOMA-IR) values {HOMA = [(fasting insulin, IU/ml) × (fasting glucose, mmol/L)]/22.5}, which attest of the sensitivity to insulin, therefore of the metabolic health of the participants (Antuna-Puente et al., 2011). All tests were performed during the follicular phase of the participants’ menstrual cycle to avoid the confounding effects of the hormonal surges during the other phases on circulating cytokine levels. Aerobic fitness was assessed through maximal oxygen consumption (VO_2_max) incremental test on a cycle ergometer as in Costford et al. (2010), and VO_2_max was reached if the respiratory exchange ratio increased to 1.10 or higher and the participant’s heart rate increased to within 10 beats of the age-predicted maximum [208 - (0.7×age)] or the rate of perceived exertion was 17 or over on a scale of 6–20.

### Myokine Quantification

Secreted protein acidic rich in cysteine (also known as osteonectin) and FGF21 were quantified by single plex assays from MesoScale Discovery (R-plex; MSD, Maryland, United States), whereas IL-6, IL-8, IL-10, IL-13, IL-15, and IL-18 were quantified by multiplex assay (U-plex; MSD) in plasma obtained from venous blood samples taken from the arm of the participants. Intra-assay coefficients of variations for the standards for SPARC, FGF21, and the multiplex were 10.00, 8.02, and 8.28%, respectively. Samples were measured only once; therefore, their intra- and interassay coefficients of variations cannot be calculated. All antibodies, with the exception of SPARC, were validated for target specificity. Information can be found on the datasheet of the different U-plex antibody sets^1^ in the Supplementary Table S2.

### Statistical Analyses

Data are presented as means ± standard deviation (SD). Anthropometric measures were analyzed by unpaired *t*-test or Welch’s *t*-test (depending on the standard error values), while all myokine quantifications were analyzed by two-way ANOVA with repeated measures or mixed model analysis (when samples from certain time points were missing) along with Dunnett’s multiple comparison, as *post hoc* tests and area under the curve (AUC) of all time points were assessed and were analyzed by unpaired *t*-test or Welch’s *t*-test. All statistical analyses were performed with Prism 8 software (San Diego, CA, United States). A *p* ≤ 0.05 was considered significant.

## Results

### Characteristics of the Participants

The characteristics of the participants are presented in Table 1. No statistical differences were found between the two groups that performed the acute bout regarding age, blood glucose, insulin, insulin sensitivity (HOMA-IR), and VO_2_max, but the BMI (*p* < 0.05), the fat mass (*p* < 0.05), and the total lean mass (*p* < 0.01) of the women from the obese group were significantly higher than the non-obese group. The HOMA-IR values in the two groups of non-obese or obese sedentary individuals suggest that these participants were insulin resistant.

**TABLE 1:** Participant characteristics of women having completed a single, acute bout of moderate intensity continuous exercise.

**Group**	***n***	**Age (years)**	**BMI (kg/m^2^)**	**Fat mass (kg)**	**Lean mass (kg)**	**Glucose (mmol/L)**	**Insulin (μIU/ml)**	**HOMA-IR**	**HbA1c (%)**	**VO_2_max (ml/min)**
Non-obese	5	28.80 ± 8.41	27.38 ± 1.40	27.69 ± 4.95	38.16 ± 4.54	5.01 ± 0.43	14.04 ± 7.80	3.21 ± 1.92	–	1,606 ± 305
Obese	6	30.00 ± 6.93	35.43 ± 5.44^†^	45.93 ± 12.43^†^	50.22 ± 2.84^††^	5.02 ± 0.32	14.48 ± 4.96	3.28 ± 1.30	5.73 ± 0.33	1,817 ± 282

*Data are means ±SD. ^†^*p* < 0.05 and ^††^*p* < 0.01 in comparison to non-obese group.*

### Time Course of Plasma Myokine Concentrations in Response to Acute Exercise

**FIGURE 1:**
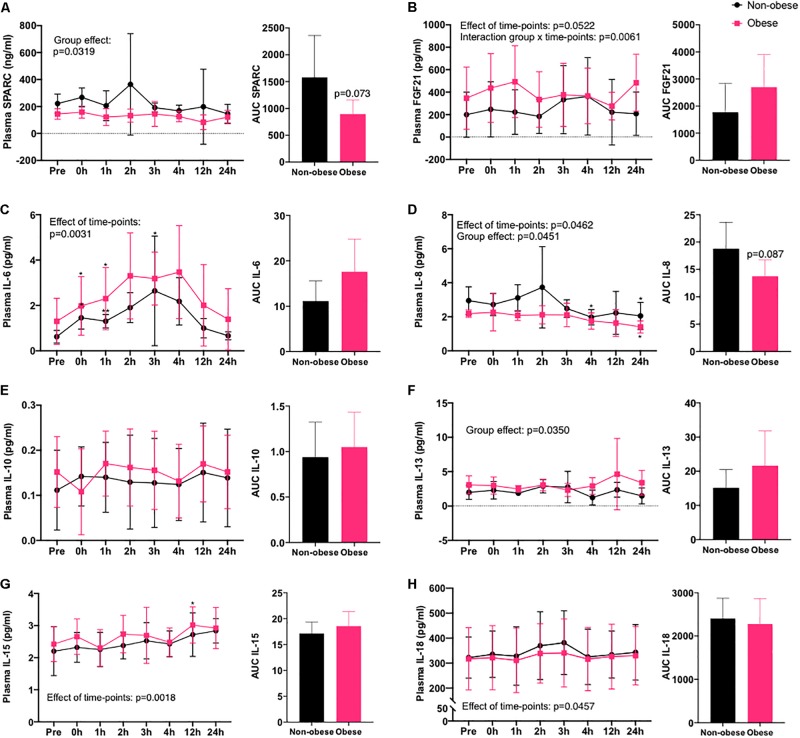
Myokines in circulation after an acute bout of exercise in women with or without obesity. Time course of concentrations of myokines in plasma of non-obese and obese women following 60 min of cycling at 60% VO_2_peak at eight different time points (pre-, immediately after (0 h), 1, 2, 3, 4, 12, and 24 h into recovery) and AUC of all time points. **(A)** SPARC, **(B)** FGF21, **(C)** IL-6, **(D)** IL-8, **(E)** IL-10, **(F)** IL-13, **(G)** IL-15, and **(H)** IL-18. Non-obese group, *n* = 5 (black circles) and obese group, *n* = 6 (pink squares). *p < 0.05, **p < 0.01 in comparison to pre-exercise (pre), effect of time points relates to variations after the exercise bout and group effect relates to BMI classifications.

In response to an acute cycling bout, FGF21, IL-6, IL-8, IL-15, and IL-18 concentrations were significantly altered in plasma of both obese and non-obese women subjects over 24 h after the exercise (Figure 1). For IL-6 and IL-18, these myokines increased transiently after the exercise bout and returned to pre-exercise values in the circulation within 24 h into the recovery period in both subject groups. On the contrary, IL-8 levels in plasma decreased gradually over the 24 h following the exercise bout, reaching significance at the 4 and 24 h time point, while IL-15 levels increased continuously to reach levels higher than basal after 12 h into recovery. No significant effect of the exercise bout was detected on plasma levels of SPARC, IL-10, and IL-13 over the 24 h of blood collection. IL-6 was the only myokine significantly increased in plasma immediately after the exercise bout, and the absolute increase relative to baseline was similar in both groups. At all time points (basal levels and levels in response to the exercise bout), SPARC and IL-8 levels in plasma were lower in the obese group, while IL-13 levels were higher in obese women in comparison to non-obese subjects. Quantification of the AUC indicated a trend toward decreased overall IL-8 (*p* = 0.087) and SPARC (*p* = 0.073) in the obese group. Finally, a significant interaction between obesity status and the variation in FGF21 levels following the exercise bout was detected, suggesting the rise in levels of this myokine in plasma after acute exercise was earlier in the participants with obesity, and they remained elevated after 24 h rather than returning to basal as in the non-obese group.

## Discussion

This study focused on the comparison in plasma myokine levels in women with obesity vs. women without obesity after a bout of moderate-intensity continuous cycling. Plasma myokines were assessed over a 24-h time course following the acute exercise to account for the different timing of secretion of individual peptides. Most of the myokines measured were regulated by acute exercise (FGF21, IL-6, IL-8, IL-15, and IL-18), and some were released at different levels in plasma over the 24 h in the group of women with obesity in comparison to the non-obese group (SPARC, IL-8, and IL-13). The regulation of plasma FGF21 following acute exercise was different in the non-obese group in comparison to the obese group.

### IL-6

Regarding IL-6, the magnitude and the time course of the increase in plasma IL-6 following the exercise bout was similar to a previous study in which normal-weight and obese subjects had completed 30 min of moderate-intensity aerobic exercise (Slusher et al., 2015). In muscle biopsies from healthy individuals taken at different time points following a running bout, IL-6 mRNA expression was found to be induced immediately by exercise (Louis et al., 2007). These results align with the variations in circulating IL-6 levels that we observed over the 24 h following the acute exercise bout completed by our sedentary participants. As reviewed by Lombardi et al., acute increases in IL-6 secretion are better detected in plasma than in serum, further highlighting the relevance of our results (Lombardi et al., 2017).

### IL-8

No increase in plasma IL-8 was detected after the cycling bout, although others found increased muscle IL-8 expression after a running exercise (Louis et al., 2007). This suggests that the increased expression of IL-8 during muscle contraction might result in the release of the protein within the muscle *interstitium*. These results support the hypothesis of Nielsen and colleagues regarding the potential autocrine role of IL-8 in muscle metabolic adaptations to physical activity (Nielsen and Pedersen, 2007). On the other hand, IL-8 levels are higher in serum than in plasma (Lombardi et al., 2017). Perhaps, differences in circulating IL-8 levels after the exercise bout would have been more significant in serum than in plasma, as this myokine seems to be released primarily in that fraction of the blood. Circulating resting and exercise-induced IL-8 was reduced in participants from the obese group, which could potentially be explained by their body composition, as women in the non-obese group had significantly less total lean and fat mass. Since myokines were measured solely in the circulation, we cannot hypothesize on the effect of either tissue to explain this difference between the study groups.

### IL-10

To our knowledge, no studies have been published on the effect of exercise on circulating levels of IL-10 in metabolically healthy subjects. On the other hand, in support of our findings, others found no effect of acute exercise on plasma IL-10 levels in patients with T2D (Korb et al., 2018). Since this myokine has been shown to have a positive effect on skeletal muscle metabolism (Hong et al., 2009; Dagdeviren et al., 2016), perhaps its mechanism of action in response to exercise is autocrine and unrelated to circulating concentrations. This hypothesis would support our findings, as well as those of others, regarding the lack of response to an acute bout of exercise in circulating IL-10. A more molecular analysis of the signaling mechanisms in muscle *per se* would be required to elucidate the effects of exercise on muscle IL-10 secretion and/or expression.

### IL-13

Concerning IL-13, plasma levels were higher in the obese in comparison to the non-obese group at rest and in response to the acute bout of exercise. Others have shown that serum and muscle IL-13 is reduced in non-obese patients with T2D, a common comorbidity of obesity (Jiang et al., 2013). Further investigations would be required to assess how obesity and/or T2D affect circulating IL-13. Our results for plasma IL-13 in response to acute exercise showed great variability, preventing us to draw any clear conclusion. In many samples, IL-13 was undetected, which affected the power to detect any significant differences between groups or in response to exercise.

### IL-15

Our results demonstrated that IL-15 circulating levels were not immediately increased after the bout of cycling, although this myokine was previously found to be higher in plasma after an acute bout of cycling exercise (Sabaratnam et al., 2018). In their study, plasma IL-15 levels returned to lower levels than baseline 3 h into recovery, while we show a steady increase as far out as 12 h. These discrepancies could be explained by the fact that our participants were women, while theirs were men. On another note, an acute running bout in healthy participants induced a gradual increase in muscle IL-15 mRNA expression over the 24 h following exercise, aligning with our results (Louis et al., 2007). Furthermore, IL-15 can be found in circulation in its free form or complexed to a soluble form of the alpha subunit of the IL-15 receptor (sIL-15Ralpha), which may alter IL-15 activity depending on the target cells as discussed in Nadeau and Aguer (2019). The kit used in the study mentioned previously shows ∼21% cross-reactivity with the complexed form of IL-15 according to the manufacturer (Human IL-15 Quantikine ELISA Kit, R&D Systems Inc., MN, United States), while the one we used (MSD) showed average reactivity at physiologically relevant doses (1.1–17.2 pg/ml) of ∼38% (Supplementary Figure S1). Since the half-life of free IL-15 is relatively short (∼30 min) and increases when IL-15 is complexed to sIL-15Ralpha (20–25 h), it is likely that the increase we observed after acute exercise lasting into the recovery period was due to the recognition of the complex, as discussed in Nadeau and Aguer (2019). Further studies would be required to better understand the mechanisms of free IL-15 and IL-15/sIL-15Ralpha complex secretion in response to exercise. Moreover, resting plasma IL-15 levels were found to be lower in physically active individuals in comparison to sedentary subjects and even higher in sedentary obese patients (Perez-Lopez et al., 2018). Therefore, we anticipated that the group of women with obesity would show higher plasma IL-15, but we found no significant differences between the two groups. Since Perez-Lopez and colleagues used the kit mentioned previously to detect IL-15, we again speculate that the differences in our results stem from the detection of the complexed form of IL-15 in our assay. Finally, the matrix does not affect IL-15 recovery when measuring this myokine in the circulation, which suggests that the results would have been similar if serum samples had been analyzed (Lombardi et al., 2017).

### SPARC

Circulating SPARC levels were unaffected immediately after an acute exercise bout, although others found an increase in serum SPARC in rats after acute exercise that mirrored increased muscle protein content (Matsuo et al., 2017). The same group also found a transient increase in serum SPARC in humans after a bout of high-intensity interval exercise or moderate-intensity continuous exercise. Data relating to preanalytical parameters for the quantification of SPARC in the circulation are lacking; it is possible, however, that variations in SPARC levels after exercise are better detected in serum than plasma. Nevertheless, others showed that a single 20-s “all-out” cycling sprint induced a significant rise in serum SPARC but that correcting circulating SPARC levels with the exercise-induced changes in plasma volume negated this effect (Songsorn et al., 2017). This finding highlights the importance of measuring hematocrit and hemoglobin levels pre- and postexercise to verify that the potential exercise-induced alterations in circulating protein concentrations are not due to the changes in plasma volume after exercise (Alis et al., 2015; Dill and Costill, 1974).

### FGF21

FGF21 is primarily expressed and produced by the liver (Nishimura et al., 2000), but its expression is also induced in skeletal muscle during exercise, and serum FGF21 increases following an aerobic exercise bout (Tanimura et al., 2016). We observed no significant increase in plasma FGF21 after the cycling bout in either groups, which confirms the findings of others regarding changes in plasma or serum FGF21 after exercise in patients with obesity, but contradicts the results obtained in healthy participants (Slusher et al., 2015; Sabaratnam et al., 2018). In normal weight (BMI = 18.5–24.9 kg/m^2^) men and women, FGF21 was found to be acutely increased in plasma after exercise; however, since our sedentary group had an average BMI classified as overweight (25–29.9 kg/m^2^), perhaps the response in FGF21 secretion was altered similarly as in patients with obesity. Of note, the average circulating FGF21 levels detected in our groups were slightly elevated compared to values from previous studies. Hansen et al. (2015) compared the effect of exercise on FGF21 release from the liver in comparison to leg muscle and found no significant release of this protein in the circulation from the skeletal muscle, whereas the splanchnic bed secreted up to fourfold more FGF21 after acute exercise. These findings confirm the hypothesis that skeletal muscle is not the main source of circulating FGF21, even during exercise.

### Short-Comings and Limitations

Our study design did not allow for the measurement of myokine secretion directly from skeletal muscle, as similar peptides can be secreted by other tissues (e.g., adipose tissue, liver, brain, etc.) (Garneau and Aguer, 2019; Weigert et al., 2019). Therefore, we cannot say for certain that all of the measured peptides originated from skeletal muscle in response to exercise. However, we found no correlations between any of the myokines and fat mass or BMI (data not shown), suggesting that adipose tissue is likely not responsible for any of the observed effects of acute exercise on variations in circulating myokines. Nevertheless, it is possible that other organs, such as the liver, might have contributed to the changes in circulating peptides levels following the cycling bout, as discussed regarding FGF21. A solution to this problem is the use of skeletal muscle biopsies to quantify myokine secretion directly from the muscle (Garneau and Aguer, 2019). However, myokines measured in skeletal muscle cannot be assumed to originate solely from muscle secretion, as the peptides could be secreted by other tissues to act on skeletal muscle during exercise. In addition, increased levels of a peptide in muscle after exercise does not necessarily translate into its release in the circulation.

Although some of the myokines measured are more stable and/or better detected in plasma, others are ideally quantified in serum. Therefore, changes in myokine secretion in the circulation might go undetected by analyses performed solely in plasma samples. Another limitation of our analyses consists in the single measurement of every sample, preventing us to calculate intra- or interassay coefficients of variation for all myokines. In addition, exercise modalities might be great contributors to the outcome on muscle signaling. The combination of aerobic and resistance exercises confers more significant changes in glucose homeostasis and muscle substrate metabolism in patients with T2D and is therefore of great interest regarding muscle signaling adaptations to exercise (Church et al., 2010; Sparks et al., 2013). Similarly, interval exercise of elevated intensity such as Tabata exercises or sprint intervals has been shown to stimulate myokine release acutely in the circulation (Harnish and Sabo, 2016). Consequently, the plasma concentrations of myokines in the 24 h following an exercise bout might evolve differently as a function of the exercise modality performed.

Another confounding effect in our study design that could have affected the measurement of certain myokines that are also cytokines (the interleukins) is the use of a venous catheter for blood collection. This method was chosen, as it is less invasive and stressful than repetitive venipuncture for subjects undergoing eight blood draws over 24 h. However, others have shown that this method of blood sampling could induce local inflammation and affect levels of circulating cytokines to a greater extent than venipuncture (Dixon et al., 2009). In addition, it has been shown that differences in plasma volume can affect the analysis of exercise-induced circulating myokine variations (Songsorn et al., 2017). Since we did not have values for hematocrit and hemoglobin for the time points following the cycling bout, we were unable to measure plasma volume variations as a function of time during the recovery period and therefore could not correct circulating myokine levels to variations in blood composition (Dill and Costill, 1974). As we did not detect an increase in all the myokines measured in plasma following acute exercise, it is likely, nonetheless, that differences in plasma volume did not cause erroneous conclusions regarding exercise-induced myokines. Moreover, interindividual variability is a major source of concern in observing the muscle signaling adaptations to acute or chronic exercise (He et al., 2018), and this could explain the impossibility to find an effect of acute exercise for certain peptides that we measured in plasma. Likewise, since our sample size was very small, some significant effects of exercise might have been undetected. This factor is the greatest limitation of our study. Regardless, we were able to find interesting effects of both acute exercise and obesity status on circulating target myokines among our study groups. Another limitation of our study is the fact that only women were represented, but this feature can also be considered positive, as women are generally underrepresented in the literature regarding exercise metabolism and muscle signaling.

### Conclusion

Our findings relating to the time course of plasma myokine levels following acute exercise depending on BMI classification (obese or non-obese) shed light on the mechanisms of endocrine signaling after physical activity. We found a regulation of IL-6, IL-8, IL-15, IL-18, and FGF21 in plasma by exercise, while obesity status increased IL-13 and decreased IL-8 and SPARC in the circulation. The next step would be to assess the effects of acute exercise on muscle signaling directly in these two groups, for example, by measuring myokine secretion from muscle biopsies harvested before and after the exercise bout. In this sense, it would be possible to compare signaling adaptations in participants with or without obesity in the circulation with the ones observed locally in skeletal muscle and potential correlations or distinctions between myokine regulation at both levels could be made as a function of body composition (i.e., BMI).

## Data Availability Statement

The raw data supporting the conclusions of this article will be made available by the authors, without undue reservation, to any qualified researcher.

## Ethics Statement

The studies involving human participants were reviewed and approved by the Institutional Review Board at AdventHealth Orlando. The Institutional Review Board at AdventHealth Orlando approved this research for ethical standards, scientific merit, and regulatory compliance. The Office of Research Administration provided support and oversight to ensure the integrity of this research at AdventHealth Orlando. ClinicalTrials.org Identifier: NCT01911091. The patients/participants provided their written informed consent to participate in this study.

## Author Contributions

LS and SS provided laboratories of samples. CA and LG performed sample analyses, data collection, and analyses at EM’s laboratory facilities. All authors contributed to the review of the manuscript.

## Conflict of Interest

SS, SP, and LS declare that this study received funding from Takeda Pharmaceuticals Inc. None of the funding sources played a role in the collection, analysis, or interpretation of the data or in the decision to submit the manuscript for publication. The remaining authors declare that the research was conducted in the absence of any commercial or financial relationships that could be construed as a potential conflict of interest.

## Footnotes

1 https://www.mesoscale.com/
