# Supplementary figures and images for: Plasma Myokine Concentrations After Acute Exercise in Non-obese and Obese Sedentary Women
Source: Front Physiol. 2020 Feb 18;11:18. doi: 10.3389/fphys.2020.00018 (PMC7040180; doi:10.3389/fphys.2020.00018)

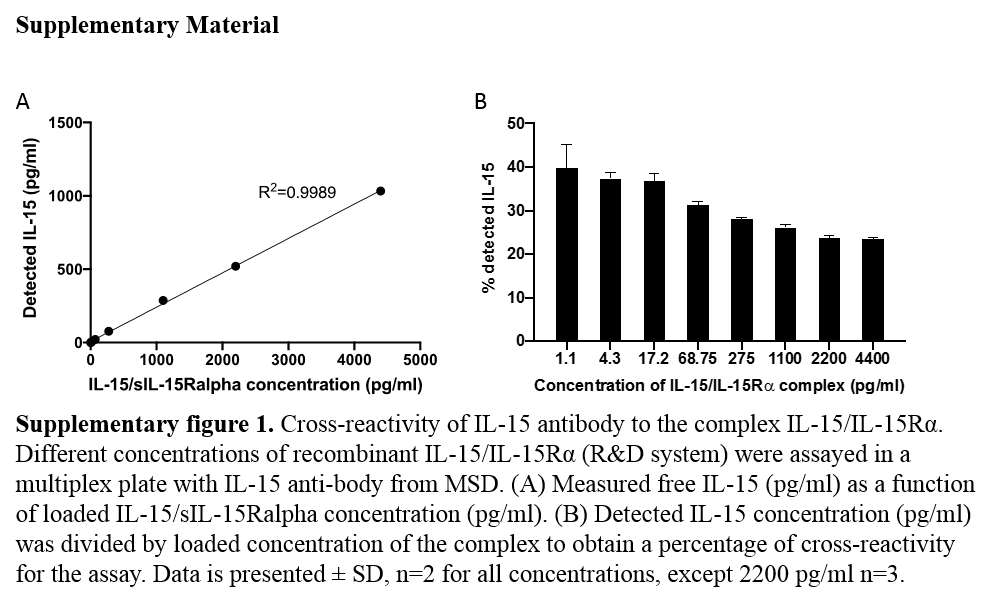

Supplement: Supplementary file 1 [file Image_1.TIF]
