# Supplementary material for: Plasma Myokine Concentrations After Acute Exercise in Non-obese and Obese Sedentary Women
Source: Front Physiol. 2020 Feb 18;11:18. doi: 10.3389/fphys.2020.00018 (PMC7040180; doi:10.3389/fphys.2020.00018)
Supplement: Supplementary file 2 [file Table_1.DOCX]

**Supplementary Materials and Methods.**

Direct links to datasheets of all antibodies used:

- IL-6: <https://www.mesoscale.com/~/media/files/data%20sheets/ds%20u-plex%20human%20il-6.pdf>
- IL-8: <https://www.mesoscale.com/~/media/files/data%20sheets/ds%20u-plex%20human%20il-8.pdf>
- IL-10: <https://www.mesoscale.com/~/media/files/data%20sheets/ds%20u-plex%20human%20il-10.pdf>
- IL-13: <https://www.mesoscale.com/~/media/files/data%20sheets/ds%20u-plex%20human%20il-13.pdf>
- IL-15: <https://www.mesoscale.com/~/media/files/data%20sheets/ds%20u-plex%20human%20il-15.pdf>
- IL-18: <https://www.mesoscale.com/~/media/files/data%20sheets/ds%20u-plex%20human%20il-18.pdf>
- FGF21: <https://www.mesoscale.com/~/media/files/data%20sheets/ds-u-plex-human-fgf-21.pdf>
- SPARC: <https://www.mesoscale.com/~/media/files/data%20sheets/ds%20r-plex%20human%20osteonectin.pdf>
