# Supplementary material for: Plasma Myokine Concentrations After Acute Exercise in Non-obese and Obese Sedentary Women
Source: Front Physiol. 2020 Feb 18;11:18. doi: 10.3389/fphys.2020.00018 (PMC7040180; doi:10.3389/fphys.2020.00018)
Supplement: Supplementary file 3 [file Table_2.DOCX]

**Supplementary Table 1. Spike recovery and specificity of the antibodies used in the MesoScale Discovery kits used.** Information is from [**https://www.mesoscale.com/**](https://www.mesoscale.com/)

| Analytes | Spike recovery in plasma Average % recovery (% recovery range) | | | Specificity | |
| --- | --- | --- | --- | --- | --- |
|  | High | Mid | Low | Analytes tested for nonspecific binding | % of nonspecific binding = (nonspecific signal / specific signal) x 100  . |
| IL-6 | 105 (98-117) | 108 (104-113) | 97 (72-109) | BAFF, BDNF, β-NGF, C-Peptide, CTACK, ENA-78, Eotaxin, Eotaxin-2, Eotaxin 3, EPO, FGF-21, FGF-23, FLT3L, Fractalkine, FSH, G-CSF, Ghrelin (octanoylSer3), Desghrelin, GIP (1-42), GIP (3-42), GLP-1 (7-36), GLP-1 (9-36), Glucagon, GM-CSF, GRO-α, I-309, IFN-α2a, IFN-β, IFN-γ, IL-1α, IL-1β, IL-1RA, IL-2, IL-2Rα, IL-3, IL-4, IL-5, IL-7, IL-8, IL-9, IL-10, IL-12/IL-23p40, IL-12p70, IL-13, IL-15, IL-16, IL-17A, IL-17A/F, IL-17B, IL-17C, IL-17D, IL-17E/IL-25, IL-17F, IL-18, IL-21, IL-22, IL-23, IL-27, IL-29/IFN-λ1, IL-31, IL-33, Insulin, IP-10, I-TAC, Leptin, LH, MCP-1, MCP-2, MCP-3, MCP-4, M-CSF, MDC, MIF, MIP-1α, MIP-1β, MIP-3α, MIP-3β, MIP-5, PP, Proinsulin (25-110), PYY (3-36), SDF-1α, TARC, TNF-α, TNF-β, TPO, TRAIL, TSLP, VEGF-A, YKL-40 | < 0.5% |
| IL-8 | 120 (111-126) | 120 (116-127) | 107 (80-120) | BAFF, BDNF, β-NGF, C-Peptide, CTACK, ENA-78, Eotaxin, Eotaxin-2, Eotaxin 3, EPO, FGF-21, FGF-23, FLT3L, Fractalkine, FSH, G-CSF, Ghrelin (octanoylSer3), Desghrelin, GIP (1-42), GIP (3-42), GLP-1 (7-36), GLP-1 (9-36), Glucagon, GM-CSF, GRO-α, I-309, IFN-α2a, IFN-β, IFN-γ, IL-1α, IL-1β, IL-1RA, IL-2, IL-2Rα, IL-3, IL-4, IL-5, IL-6, IL-7, IL-9, IL-10, IL-12/IL-23p40, IL-12p70, IL-13, IL-15, IL-16, IL-17A, IL-17A/F, IL-17B, IL-17C, IL-17D, IL-17E/IL-25, IL-17F, IL-18, IL-21, IL-22, IL-23, IL-27, IL-29/IFN-λ1, IL-31, IL-33, Insulin, IP-10, I-TAC, Leptin, LH, MCP-1, MCP-2, MCP-3, MCP-4, M-CSF, MDC, MIF, MIP-1α, MIP-1β, MIP-3α, MIP-3β, MIP-5, PP, Proinsulin (25-110), PYY (3-36), SDF-1α, TARC, TNF-α, TNF-β, TPO, TRAIL, TSLP, VEGF-A, YKL-40 | < 0.5% |
| IL-10 | 84 (41-107) | 97 (44-122) | 86 (40-125) | BAFF, BDNF, β-NGF, C-Peptide, CTACK, ENA-78, Eotaxin, Eotaxin-2, Eotaxin 3, EPO, FGF-21, FGF-23, FLT3L, Fractalkine, FSH, G-CSF, Ghrelin (octanoylSer3), Desghrelin, GIP (1-42), GIP (3-42), GLP-1 (7-36), GLP-1 (9-36), Glucagon, GM-CSF, GRO-α, I-309, IFN-α2a, IFN-β, IFN-γ, IL-1α, IL-1β, IL-1RA, IL-2, IL-2Rα, IL-3, IL-4, IL-5, IL-6, IL-7, IL-8, IL-9, IL-12/IL-23p40, IL-12p70, IL-13, IL-15, IL-16, IL-17A, IL-17A/F, IL-17B, IL-17C, IL-17D, IL-17E/IL-25, IL-17F, IL-18, IL-21, IL-22, IL-23, IL-27, IL-29/IFN-λ1, IL-31, IL-33, Insulin, IP-10, I-TAC, Leptin, LH, MCP-1, MCP-2, MCP-3, MCP-4, M-CSF, MDC, MIF, MIP-1α, MIP-1β, MIP-3α, MIP-3β, MIP-5, PP, Proinsulin (25-110), PYY (3-36), SDF-1α, TARC, TNF-α, TNF-β, TPO, TRAIL, TSLP, VEGF-A, YKL-40 | < 0.5% |
| IL-13 | 106 (74-112) | 118 (81-159) | 109 (77-145) | BAFF, BDNF, β-NGF, C-Peptide, CTACK, ENA-78, Eotaxin, Eotaxin-2, Eotaxin 3, EPO, FGF-21, FGF-23, FLT3L, Fractalkine, FSH, G-CSF, Ghrelin (octanoylSer3), Desghrelin, GIP (1-42), GIP (3-42), GLP-1 (7-36), GLP-1 (9-36), Glucagon, GM-CSF, GRO-α, I-309, IFN-α2a, IFN-β, IFN-γ, IL-1α, IL-1β, IL-1RA, IL-2, IL-2Rα, IL-3, IL-4, IL-5, IL-6, IL-7, IL-8, IL-9, IL-10, IL-12/IL-23p40, IL-12p70, IL-15, IL-16, IL-17A, IL-17A/F, IL-17B, IL-17C, IL-17D, IL-17E/IL-25, IL-17F, IL-18, IL-21, IL-22, IL-23, IL-27, IL-29/IFN-λ1, IL-31, IL-33, Insulin, IP-10, I-TAC, Leptin, LH, MCP-1, MCP-2, MCP-3, MCP-4, M-CSF, MDC, MIF, MIP-1α, MIP-1β, MIP-3α, MIP-3β, MIP-5, PP, Proinsulin (25-110), PYY (3-36), SDF-1α, TARC, TNF-α, TNF-β, TPO, TRAIL, TSLP, VEGF-A, YKL-40 | < 0.5% |
| IL-15 | 123 (110-135) | 110 (104-114) | 111 (99-118) | BAFF, BDNF, β-NGF, C-Peptide, CTACK, ENA-78, Eotaxin, Eotaxin-2, Eotaxin 3, EPO, FGF-21, FGF-23, FLT3L, Fractalkine, FSH, G-CSF, Ghrelin (octanoylSer3), Desghrelin, GIP (1-42), GIP (3-42), GLP-1 (7-36), GLP-1 (9-36), Glucagon, GM-CSF, GRO-α, I-309, IFN-α2a, IFN-β, IFN-γ, IL-1α, IL-1β, IL-1RA, IL-2, IL-2Rα, IL-3, IL-4, IL-5, IL-6, IL-7, IL-8, IL-9, IL-10, IL-12/IL-23p40, IL-12p70, IL-13, IL-16, IL-17A, IL-17A/F, IL-17B, IL-17C, IL-17D, IL-17E/IL-25, IL-17F, IL-18, IL-21, IL-22, IL-23, IL-27, IL-29/IFN-λ1, IL-31, IL-33, Insulin, IP-10, I-TAC, Leptin, LH, MCP-1, MCP-2, MCP-3, MCP-4, M-CSF, MDC, MIF, MIP-1α, MIP-1β, MIP-3α, MIP-3β, MIP-5, PP, Proinsulin (25-110), PYY (3-36), SDF-1α, TARC, TNF-α, TNF-β, TPO, TRAIL, TSLP, VEGF-A, YKL-40 | < 0.5% |
| IL-18 | 92 (73-102) | 92 (74-100) | 91 (81-97) | BAFF, BDNF, β-NGF, C-Peptide, CTACK, ENA-78, Eotaxin, Eotaxin-2, Eotaxin 3, EPO, FGF-21, FGF-23, FLT3L, Fractalkine, FSH, G-CSF, Ghrelin (octanoylSer3), Desghrelin, GIP (1-42), GIP (3-42), GLP-1 (7-36), GLP-1 (9-36), Glucagon, GM-CSF, GRO-α, I-309, IFN-α2a, IFN-β, IFN-γ, IL-1α, IL-1β, IL-1RA, IL-2, IL-2Rα, IL-3, IL-4, IL-5, IL-6, IL-7, IL-8, IL-9, IL-10, IL-12/IL-23p40, IL-12p70, IL-13, IL-15, IL-16, IL-17A, IL-17A/F, IL-17B, IL-17C, IL-17D, IL-17E/IL-25, IL-17F, IL-21, IL-22, IL-23, IL-27, IL-29/IFN-λ1, IL-31, IL-33, Insulin, IP-10, I-TAC, Leptin, LH, MCP-1, MCP-2, MCP-3, MCP-4, M-CSF, MDC, MIF, MIP-1α, MIP-1β, MIP-3α, MIP-3β, MIP-5, PP, Proinsulin (25-110), PYY (3-36), SDF-1α, TARC, TNF-α, TNF-β, TPO, TRAIL, TSLP, VEGF-A, YKL-40 | < 0.5% |
| FGF21 | 92 (73-102) | 92 (88-102) | 91 (95-103) | AFF, BDNF, β-NGF, C-Peptide, CTACK, ENA-78, Eotaxin, Eotaxin-2, Eotaxin 3, EPO, FGF-23, FLT3L, Fractalkine, FSH, G-CSF, Ghrelin (octanoylSer3), Desghrelin, GIP (1-42), GIP (3-42), GLP-1 (7-36), GLP-1 (9-36), Glucagon, GM-CSF, GRO-α, I-309, IFN-α2a, IFN-β, IFN-γ, IL-1α, IL-1β, IL-1RA, IL-2, IL-2Rα, IL-3, IL-4, IL-5, IL-6, IL-7, IL-8, IL-9, IL-10, IL-12/IL-23p40, IL-12p70, IL-13, IL-15, IL-16, IL-17A, IL-17A/F, IL-17B, IL-17C, IL-17D, IL-17E/IL-25, IL-17F, IL-18, IL-21, IL-22, IL-23, IL-27, IL-29/IFN-λ1, IL-31, IL-33, Insulin, IP-10, I-TAC, Leptin, LH, MCP-1, MCP-2, MCP-3, MCP-4, M-CSF, MDC, MIF, MIP-1α, MIP-1β, MIP-3α, MIP-3β, MIP-5, PP, Proinsulin (25-110), PYY (3-36), SDF-1α, TARC, TNF-α, TNF-β, TPO, TRAIL, TSLP, VEGF-A, YKL-40 | < 0.5% |
| SPARC | N/A | N/A | N/A | N/A | N/A |
